# *CCP*4 Cloud for structure determination and project management in macromolecular crystallography

**DOI:** 10.1107/S2059798322007987

**Published:** 2022-08-30

**Authors:** Eugene Krissinel, Andrey A. Lebedev, Ville Uski, Charles B. Ballard, Ronan M. Keegan, Oleg Kovalevskiy, Robert A. Nicholls, Navraj S. Pannu, Pavol Skubák, John Berrisford, Maria Fando, Bernhard Lohkamp, Marcin Wojdyr, Adam J. Simpkin, Jens M. H. Thomas, Christopher Oliver, Clemens Vonrhein, Grzegorz Chojnowski, Arnaud Basle, Andrew Purkiss, Michail N. Isupov, Stuart McNicholas, Edward Lowe, Josep Triviño, Kevin Cowtan, Jon Agirre, Daniel J. Rigden, Isabel Uson, Victor Lamzin, Ivo Tews, Gerard Bricogne, Andrew G. W. Leslie, David G. Brown

**Affiliations:** a Scientific Computing Department, Science and Technology Facilities Council UK, Didcot OX11 0FA, United Kingdom; b Structural Studies Division, MRC Laboratory for Structural Biology, Cambridge CB2 0QH, United Kingdom; c Leiden University Medical Center, 2333 ZA Leiden, The Netherlands; d European Bioinformatics Institute, Hinxton CB9 1SD, United Kingdom; e Institute of Protein Research, Pushchino 142290, Russian Federation; f Translational and Clinical Research Institute, Newcastle University, Framlington Place, Newcastle upon Tyne NE2 4HH, United Kingdom; g Biological Sciences, Institute for Life Sciences, University of Southampton, Southampton SO17 1BJ, United Kingdom; h Department of Medical Biochemistry and Biophysics, Karolinska Institutet, SE-171 77 Stockholm, Sweden; i Global Phasing Limited, Sheraton House, Castle Park, Cambridge CB3 0AX, United Kingdom; j Institute of Systems, Molecular and Integrative Biology, University of Liverpool, Liverpool L69 7ZB, United Kingdom; k School of Physics and Astronomy, University of Birmingham, Birmingham B15 2TT, United Kingdom; l European Molecular Biology Laboratory, Hamburg Unit, Notkestrasse 85, 22607 Hamburg, Germany; m Newcastle University Biosciences Institute Medical School, Newcastle upon Tyne NE2 4AX, United Kingdom; n Structural Biology Science Technology Platform, The Francis Crick Institute, 1 Midland Road, London NW1 1AT, United Kingdom; o Biosciences, University of Exeter, Stocker Road, Exeter EX4 4QD, United Kingdom; p York Structural Biology Laboratory, Department of Chemistry, University of York, York YO10 5DD, United Kingdom; q Department of Biochemistry, University of Oxford, South Parks Road, Oxford OX1 3QU, United Kingdom; r Crystallographic Methods, Institute of Molecular Biology of Barcelona (IBMB–CSIC), Barcelona Science Park, Helix Building, Baldiri Reixac 15, 08028 Barcelona, Spain; s ICREA: Institució Catalana de Recerca i Estudis Avançats, Pg. Lluis Companys 23, 08010 Barcelona, Spain; t Servier Research Institute in Croissy-sur-Seine, 125 Chemin de Ronde, 78290 Croissy, France; Institute of Integrative Biology, University of Liverpool, United Kingdom

**Keywords:** *CCP*4, *CCP*4 Cloud, macromolecular crystallography, distributed computing, data management, project management

## Abstract

Nowadays, progress in the determination of three-dimensional macromolecular structures from diffraction images is achieved partly at the cost of increasing data volumes. This is due to the deployment of modern high-speed, high-resolution detectors, the increased complexity and variety of crystallographic software, the use of extensive databases and high-performance computing. This limits what can be accomplished with personal, offline, computing equipment in terms of both productivity and maintainability. There is also an issue of long-term data maintenance and availability of structure-solution projects as the links between experimental observations and the final results deposited in the PDB. In this article, *CCP*4 Cloud, a new front-end of the *CCP*4 software suite, is presented which mitigates these effects by providing an online, cloud-based environment for crystallographic computation. *CCP*4 Cloud was developed for the efficient delivery of computing power, database services and seamless integration with web resources. It provides a rich graphical user interface that allows project sharing and long-term storage for structure-solution projects, and can be linked to data-producing facilities. The system is distributed with the *CCP*4 software suite version 7.1 and higher, and an online publicly available instance of *CCP*4 Cloud is provided by CCP4.

## Abstract 2

The paper describes *CCP*4 Cloud, an online system for macromolecular structure determination based on the *CCP*4 software suite.

## 1. Introduction

Cloud-based computation has gained increasing popularity and has rapidly become the *modus operandi* almost everywhere. The significance and advantages of distributed, server-based computing have become particularly clear owing to the recent COVID-19 pandemic, when existing trends towards remote working received a truly dramatic acceleration. The main advantages offered by cloud-based setups include (i) zero maintenance burden for end users, (ii) access to significant computational resources, (iii) efficient data logistics and (iv) a long, potentially indefinite, lifetime of the obtained results with highly efficient access, sharing and dissemination possibilities. All of these are attractive for applications in macromolecular crystallography (MX).

### 1.1. Maintenance burden

As a method, MX has seen steady and active development over the past several decades and has produced a considerable variety of computational approaches and exemplary pieces of software, allowing one to cope with all computational aspects of experimental MX studies. For example, the *CCP*4 software suite (Winn *et al.*, 2011 ▸) version 8.0 presents a user with more than 500 executable modules and requires about 6.3 GB of disk space in a minimalistic setup. Full installation requires a set of additional databases and third-party software components, which require additional maintenance and increase the disk-space demand to over 50 GB. In a distributed framework, all software and databases are stored and maintained remotely (*i.e.* in the cloud) and thus are always kept in a ready and duly updated state.

### 1.2. Computational resources

MX structure solution is a complex process, and considerable effort has been invested in its automation. Today, most structures can be solved automatically, yet the corresponding algorithms can take hours and even days to be executed on ordinary PCs. The limitations are particularly apparent if large volumes of data, as are typical for modern beamlines, need to be processed. Cloud-based computations can be parallelized on many computational nodes and thereby deliver the power of centralized high-performance computing (HPC) facilities to end users.

### 1.3. Data logistics

There are three main aspects related to data maintenance in MX: (i) the manipulation of the large volumes of data and numbers of files typical of crystallographic computing, (ii) maintaining the structure-solution workflow and presenting it in a form convenient for analysis and development, and (iii) supporting teamwork and coworking from different physical and virtual locations. Modern X-ray detectors produce significant amounts of data, reaching terabyte scales per crystal. Ideally, such volumes should either be processed where they are produced or archived, without moving the data to local devices. In the cloud, this problem can be conveniently solved by linking the data-producing facility and the computing centre. The volumes of derived and intermediate data in crystallographic projects are usually not so critically large compared with the raw data, but they may be difficult to maintain due to the large number of files and the nature of the highly iterative, trial-and-error, approach that is often exercised in practice. This makes it particularly important to present the overall workflow, including abstracted project structures and all derived data, in a clear and concise way. The problem becomes more obvious if more than one computer is used during a structure-solution project or if several researchers work on the same project. In such cases, a cloud-based setup is an elegant solution due to all projects and associated data being kept in a single instance accessible from any convenient device and geographic location.

### 1.4. Data security, safety, access and dissemination

While data security in the cloud represents a topic of continual concern and research, significant progress has been achieved in this area during the last decade (Ramachandra *et al.*, 2017 ▸). Modern centralized setups are protected from attacks on a considerably higher level than personal computing items, which in fact represent the weak link in the security system. Data in the cloud, running over, for example, the company’s internal network, are as safe as anywhere else on the company’s premises. The lifetime of data stored in the cloud is considerably longer (effectively infinite) than that usually achieved with locally maintained hardware. Equally importantly, a cloud-based setup provides efficient access to its resources, which can be conveniently exploited for archiving purposes. In addition to the final results, note that whole structure-solution projects can be archived to be provided as complementary information for scientific publishing, which is potentially useful for revising and revisiting projects, or subsequently repurposed, for example as educational material.

Distributed computing is already being used within the field of MX and cryo-EM. Some examples are as follows.

(i) EMBL Hamburg provides the *ARP*/*wARP* server (Langer *et al.*, 2008 ▸) for the automatic building of protein and nucleic acid structures, and the *Auto-Rickshaw* server (Panjikar *et al.*, 2005 ▸), which offers an extensive collection of phasing methods.
(ii) Global Phasing Ltd runs online services for their *GRADE* (geometrical restraints generation; Smart *et al.*, 2011 ▸), *STARANISO* (anisotropy of the diffraction limit and Bayesian estimation of structural amplitudes; Tickle *et al.*, 2018 ▸) and *PDBpeep* (analysis of PDB data sets; Tickle *et al.*, 2018 ▸) applications.
(iii) The *TLS* server at the University of Washington (Painter & Merritt, 2006 ▸), which identifies rigid bodies in crystal structures and parametrizes their ‘motions’.
(iv) The *PISA* server at the EBI (Krissinel & Henrick, 2007 ▸), which analyses crystal packing, macromolecular interfaces and protein oligomeric states.
(v) The *PDB-REDO* server at the Netherlands Cancer Institute (Joosten *et al.*, 2014 ▸), which optimizes crystallo­graphic structures with automated algorithms for refinement, rebuilding and validation.
(vi) The SBGrid web portal, which provides access to the *Wide Search Molecular Replacement* (Stokes-Rees & Sliz, 2010 ▸) and *Deformable Elastic Network* tools for structure refinement from low-resolution data sets (O’Donovan *et al.*, 2012 ▸).
(vii) The *FragFit* server for the prediction, visualization and selection of missing segments in protein cryo-EM density maps (Tiemann *et al.*, 2018 ▸).

In addition, CCP4’s online services (Krissinel *et al.*, 2018 ▸) comprise a collection of automated tools for the following.

(i) Molecular replacement (MR): *MrBUMP* (Keegan & Winn, 2008 ▸), *BALBES* (Long *et al.*, 2008 ▸) and *MoRDA* (Vagin & Lebedev, 2015 ▸).
(ii) Experimental phasing: *Crank*2 (Skubák & Pannu, 2013 ▸) and *SHELX* (Sheldrick *et al.*, 2012 ▸; Usón & Sheldrick, 2018 ▸).
(iii) Bias-free MR with fragments: *ARCIMBOLDO* (Millán *et al.*, 2015 ▸).
(iv) MR with *ab initio* modelling: *AMPLE* (Bibby *et al.*, 2012 ▸).
(v) MR with database searches: *SIMBAD* (Simpkin *et al.*, 2018 ▸).
(vi) Validation and analysis: *PISA* (Krissinel, 2015 ▸) and *Zanuda* (Lebedev & Isupov, 2014 ▸).

This compilation of online resources is far from complete, yet the number and variety of existing developments does suggest that cloud-based computing represents an attractive solution that is already appreciated by the MX research community.

All MX-related web services that we are aware of are designed to perform specific tasks. Even taken together, they do not form a complete system for solving MX structures, and there is no single facility for developing full MX projects online. Individual web services were not generally developed with a common framework for data exchange in mind, which is not convenient for their integration. Nevertheless, a few web services were integrated as part of the WestLife initiative (Morris *et al.*, 2019 ▸), and the *BALBES* and *MoRDa* servers at CCP4 can send phased structures to the *ARP*/*wARP* web server at EMBL Hamburg for subsequent model building, using *ad hoc* communication protocols.

In this publication, we present *CCP*4 Cloud, the new online solution for MX computation developed by the Collaborative Computational Project Number 4 in protein crystallography (CCP4 UK). This development was aimed at providing a generic, complete, distributable, highly versatile and scalable framework for performing MX computations on a set of remote servers, which would allow the creation, development and storage of full MX structure-solution projects online. Further ambitions that have been realized include a rich, ergonomic graphical user interface with the possibility of sharing projects for simultaneous teamwork, seamless access to external web resources, a framework for accommodating software not licenced by CCP4, integrated documentation and educational materials, and the abilities to link to data-producing facilities and to run locally as a conventional desktop GUI.

## 2. *CCP*4 Cloud architecture and implementation

*CCP*4 Cloud can be viewed as a set of logical components which function independently of each other and exchange data via the http(s) protocol. The components are combined into a working system by specifying the URL addresses of interacting partners in their configuration files.

There are four types of logical components in *CCP*4 Cloud: (i) Front End Server (FE), (ii) Number Cruncher (NC), (iii) CCP4 Cloud Client (CC) and (iv) User Interface (UI). The *CCP*4 Cloud configuration is defined by the number of logical components of each type in the system and their distribution over hardware hosts. Below, we briefly consider the role of each component and a few representative configuration templates.

### 2.1. Front-End Server

The Front-End Server (FE) is the central element of the system that is responsible for the overall data logistics (but not computations) in the system. The FE keeps user accounts, structure-solution projects and data, maintains connection with UIs on user machines, provides access to external data resources and web services, prepares data and metadata for running jobs on NCs, dispatches jobs to NCs and balances their load, and relays communication between the UI and any running jobs. Typically, a *CCP*4 Cloud setup has a single FE, but the system may be configured to use several FEs if necessary for coping with extra workload or providing groups of users with their own gateways.

### 2.2. Number Crunchers

Number Crunchers (NCs) are servers whose sole task is to run computational jobs. They receive input data and execution instructions from the FE(s), perform calculations on the attached back-end and push results back to the FE that sent the task. NCs can be configured to work with virtually any computational back-end, for example, a cluster queue (SGE, SLURM *etc.*), another machine with SSH access or just a shell on the NC’s host machine.

There must be at least one NC in a *CCP*4 Cloud setup. However, the system is designed to have multiple computational nodes, and can be heterogeneous in respect to the hardware platforms, operating systems and back-ends used. NCs can accept jobs from multiple FEs, making sure that the results are pushed back to the sender.

### 2.3. CCP4 Cloud Client

CCP4 Cloud Client (CC) is a special type of NC that runs on the user’s device and enables the use of *CCP*4 graphical applications with *CCP*4 Cloud: *Coot* (Emsley *et al.*, 2010 ▸), *CCP*4*mg* (McNicholas *et al.*, 2011 ▸), *DUI* (Winter *et al.*, 2018 ▸), *iMosflm* (Battye *et al.*, 2011 ▸) and *ViewHKL* (Evans & Krissinel, unpublished work). In the current version of *CCP*4, these applications cannot be executed within a browser and thus must run from a local installation of the *CCP*4 software suite. The CC provides a seamless integration of the local *CCP*4 installation with the rest of *CCP*4 Cloud, making all communication and data exchange completely automatic.

CCs are important for image-processing tasks. Typically, X-ray diffraction images have a considerable size, which makes uploading them from the user’s machine to remote servers impractical. Therefore, in addition to image-processing GUIs (*DUI*, *iMosflm* and optionally *XDSGUI*; Sparta *et al.*, 2016 ▸), the CC can also run the automatic image-processing pipeline *xia*2 (Winter, 2010 ▸) locally (note that *xia*2 can also be run in a remote NC using diffraction images uploaded by other means; for example, directly from a synchrotron beamline via a special link in the background).

The CC launcher is distributed as part of the *CCP*4 software suite. Besides starting the CC, it also starts a chosen web browser and connects to *CCP*4 Cloud automatically. This means that starting a full-featured *CCP*4 Cloud session is as easy as starting a desktop GUI.

Although very practical, CCs are optional elements of *CCP*4 Cloud. They require a local installation of the *CCP*4 software suite and so cannot be used when working from a device that cannot run *CCP*4 software (for example a tablet or smartphone). A session without a CC can be started by navigating to the *CCP*4 Cloud URL from within a web browser (including from a device without a *CCP*4 installation, such as a tablet or smartphone). This can be useful for reviewing a project, checking on the progress of a running job or launching non-GUI tasks when away from the desk. The FE server automatically detects whether a CC is running on the user’s device and enables the corresponding tasks accordingly.

### 2.4. User Interface

*CCP*4 Cloud is operated via a graphical User Interface (UI) in a common web browser running on the user’s device. The interface is presented in more detail below.

### 2.5. *CCP*4 Cloud configuration and typical deployment scenarios

Server nodes (FEs, NCs and CCs) are configured individually. They are plugged into the *CCP*4 Cloud installation by specifying the URL addresses of the communicating partners in configuration files. For example, an FE configuration file contains the URLs of the NCs that can accept jobs from that FE. This allows the *CCP*4 Cloud configuration to be modified very easily and to scale dynamically according to the current workload. NCs may be switched on/off without stopping the whole system, with the FE reallocating new jobs automatically. This mechanism is used for working with CCs, which are added automatically when the user connects to *CCP*4 Cloud from a CC-enabled device and are automatically removed from the system when the user ends their session.

**Figure 1:**
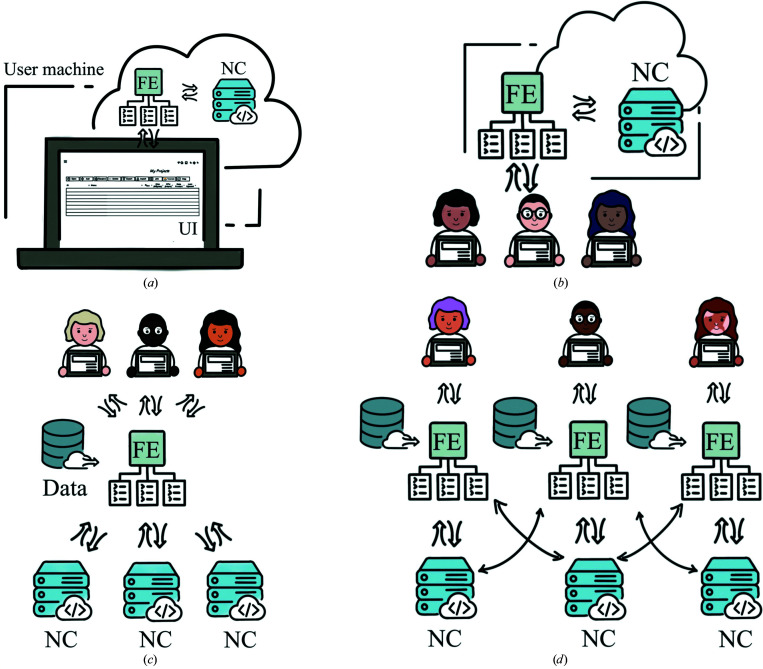
Examples of *CCP*4 Cloud configurations. FE, Front End; NC, Number Cruncher; UI, User Interface with optional CC (client-side NC); Data, access to collected X-ray diffraction data sets and other data used for MX structure determination. Arrows denote http(s) connections. (*a*) Single-host configuration, suitable for an individual working without an internet connection. (*b*) A multi-user setup using a central host machine, suitable for small to medium-sized laboratories. (*c*) Fully distributed, multi-component setup with single point of access, suitable for large facilities and research centres; it can optionally allow data acquisition from external sources. (*d*) Fully distributed setup with multiple access points. Configurations (*a*) and (*c*) work out of the box using the *CCP*4 software suite installed on the user’s machine [using the *CCP*4 Cloud setup at RAL Harwell in case (*c*)]. All configurations may be tailored to specific conditions and requirements and can be built on top of the *CCP*4 Cloud components that are included in the *CCP4* software suite. See the discussion in the text.

*CCP*4 Cloud can be configured in a number of different ways and tailored to suit a particular situation, determined mostly by the number and type of available computational back-ends, disk storage for user projects and data, and the mechanism for accessing X-ray diffraction images. Fig. 1 ▸ presents a few typical *CCP*4 Cloud configurations. In the simplest case (Fig. 1 ▸ *a*) all *CCP*4 Cloud components are placed on a single hardware host, which can be a laptop or a workstation. In this configuration, *CCP*4 Cloud runs all computational jobs on the host machine and, from the user’s perspective, acts like any other program with a GUI. This setup is suitable, for example, for users who do not wish to transmit their data to remote servers. The single-host configuration is included in *CCP*4 version 7.1 or higher and works out of the box.

Fig. 1 ▸(*b*) presents a schematic of a *CCP*4 Cloud configuration that might be suitable for medium-sized research laboratories. This configuration extends that in Fig. 1 ▸(*a*) by exposing the FE to the internet, which allows multiple users to connect to it from their own machines. Both the FE and NC run on the same machine, typically a powerful workstation or a cluster’s head node.

An example of the *CCP*4 Cloud setup maintained at the CCP4 site in Rutherford Appleton Laboratory (RAL) Harwell is shown in Fig. 1 ▸(*c*). In this type of setup the FE and NCs are placed on dedicated hardware hosts. This allows a high level of responsiveness for the users, whilst achieving a high number of running and queuing jobs. The FE has access to a dedicated file system (‘Data’ in Fig. 1 ▸ *c*) that is visible to users as a read-only ‘Cloud Storage’. This storage is different from the disk space allocated for user data and projects. The main purpose of Cloud Storage is to keep large volumes of data collected at the synchrotron and make them available for the data-processing pipeline *xia*2 in user projects. Each user can see only the part of Cloud Storage that was made accessible to them by the *CCP*4 Cloud administrator. In addition, Cloud Storage contains public areas that are usually used for keeping demo projects, tutorials and similar resources. *CCP*4 Cloud at RAL Harwell is publicly available and can be conveniently accessed via the pre-configured CC that is included as part of the *CCP*4 software suite.

The *CCP*4 Cloud configuration in Fig. 1 ▸(*d*) provides individual FEs for each user or groups of users but utilizes a common line of NCs for running jobs. This scheme is suitable in special situations, for example when user data must be kept strictly confidential, in predefined locations (such as user home directories) or protected by file-system permissions. This is achieved by running individually configured FEs under the corresponding user permissions.

### 2.6. Testing

Testing is made an integral part of *CCP*4 Cloud and is performed automatically on a nightly basis. This was found to be necessary due to the rapid development of both *CCP*4 Cloud and the underlying *CCP*4 software suite. The test system is based on the Selenium framework (Islam & Quadri, 2020 ▸), which is suitable for automatically imitating a user’s actions in a web browser, such as logging on to *CCP4* Cloud, creating projects, setting up and starting tasks. Each test represents the development of a project with a few jobs, and subsequent checks assert that all jobs complete and produce results within set tolerances. Test projects are added to the system with every new task, and most programs are tested in multiple projects featuring different contexts or exploiting different modes and combinations of parameters.

## 3. Principles of the *CCP*4 Cloud interface and project development

In *CCP*4 Cloud, work is organized into Projects. Within a Project there is a single tree of Jobs (the Project Tree), which provides a logical, rather than chronological, representation of the structure-solution pathway. This organizational concept is reinforced by using the concept of a Structure Revision, which will be described later.

### 3.1. Data objects

**Figure 2:**
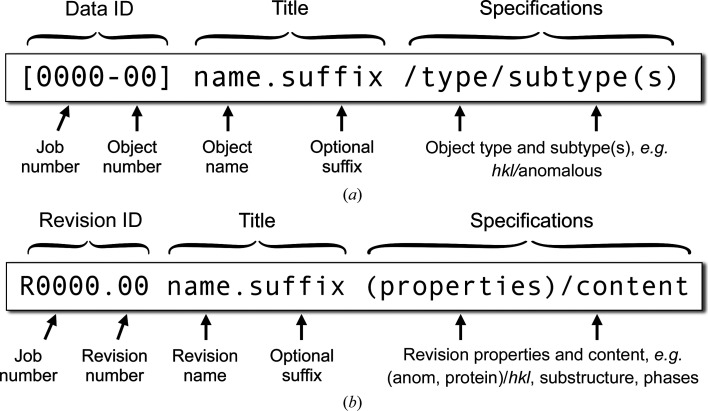
Naming conventions for data objects (*a*) and structure revisions (*b*) in *CCP*4 Cloud. All fields are generated automatically, and only ‘name’ can be overridden by the user. Suffixation is used when a job produces multiple outputs, for example for the original and inverted hands in experimental phasing.

Unlike most desktop GUIs, the *CCP*4 Cloud UI operates with data objects, rather than files. There are two main reasons for this: firstly, the *CCP*4 Cloud file system is not accessible to users, and secondly a suitable choice of data-object types simplifies the overall data logistics. As a result, specification of job input becomes very minimal and in many cases automatic. A data object may correspond to a file, but it can also represent a selected part of a file or be a composition of several files. At a low level, data objects are represented by JSON-formatted files containing references to files with all necessary metadata such as selection ranges. Once produced, the files never change and remain in the original job directories; instead, it is data objects that are communicated within the system. Interconversion between objects and files is performed automatically as an internal part of computational Jobs; this is seamlessly abstracted from the user. Whenever a data object is created on output of a Job, download links are provided for all files that it refers to. Table 1 ▸ lists the data objects currently used in *CCP*4 Cloud and their file equivalents. All data objects are named automatically and uniquely using templates shown in Fig. 2 ▸.

Jobs can see data objects produced by all their ancestors in the Project Tree, except for Structure Revisions, which can be taken only from first ancestors (*i.e.* the immediately previous Job higher up in the Project Tree, which is always unique). A Structure Revision is a special data object which provides the latest representation of the crystal (reflection and sequence data, atomic coordinates and phases) obtained at a particular point of the Project Tree. The limited scope of the visibility of Structure Revisions is necessary for keeping the Project Tree logically branched. Thus, if calculations need to be repeated with the same input data but modified parameters or using a different program, it can be performed simply by creating a new job from the point of decision (and only from that point), resulting in a new branch being created within the Project Tree.

### 3.2. Jobs

**Figure 3:**
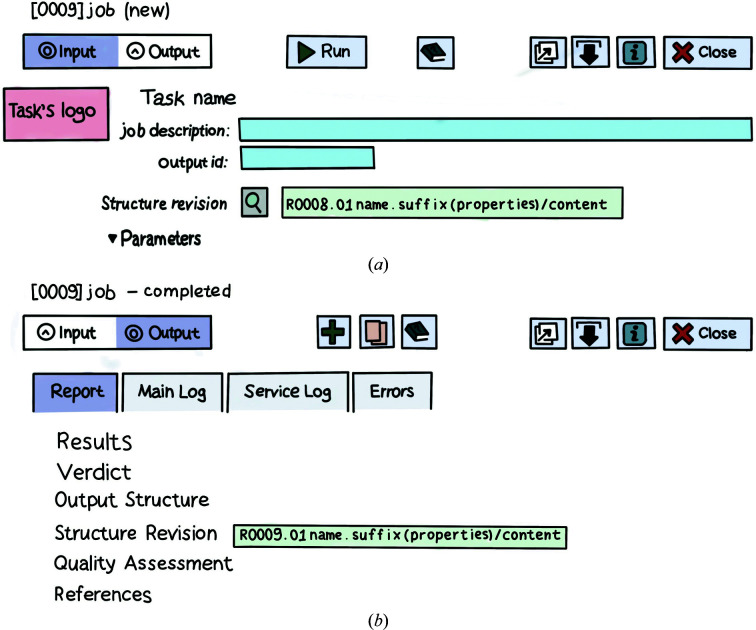
Schematic of *CCP*4 Cloud task interfaces. (*a*) input panel, (*b*) output panel (Job report).

*CCP*4 Cloud Jobs are usually centred around primary components, such as *Phaser* (McCoy *et al.*, 2007 ▸), *REFMAC* (Murshudov *et al.*, 2011 ▸), *Coot* *etc.*, and use several more programs behind the scenes to perform file-format interconversions, prepare intermediate data files and analyse results. This organization results in the user being provided with a refined set of crystallographic tasks, which is more digestible than an exhaustive and overwhelming list of software tools. Task interfaces follow a common pattern (Fig. 3 ▸ *a*): all input data objects are placed in the upper part of the interface, with task parameters below them. All input fields are given default values, including input data objects. In most cases, input data are taken from the Structure Revision that was generated by the previous Job, making the data choice automatic and unambiguous. Default values for other data objects are chosen with some heuristics and should always be checked before running a task.

The job output (Fig. 3 ▸ *b*) is placed in a dedicated tab and shows the progress of the job in real time. Real-time monitoring is useful for early termination of a job if the output suggests that an acceptable solution will not be achieved. Job reports provide a graphical representation of results, a description of any data objects created by the Job, as well as links to the in-browser graphical viewer *UglyMol* (Wojdyr, 2017 ▸), which allows quick inspection of structural models and electron-density maps. Where appropriate, Job reports include Verdict and Quality Assessment sections. Verdicts present achieved scores, such as *R* factors, completeness, number of clashes, Ramachandran outliers *etc.*, along with suggestions for the modification of job parameters and follow-up steps. Whenever a structural model is modified, a Quality Assessment is performed that utilizes a standard set of programs, including *MolProbity* (Chen *et al.*, 2010 ▸), *EDStats* (Tickle *et al.*, 1998 ▸) and *B*-factor analysis (Winn *et al.*, 2011 ▸), and presents their results in a summary table.

### 3.3. Project development

**Figure 4:**
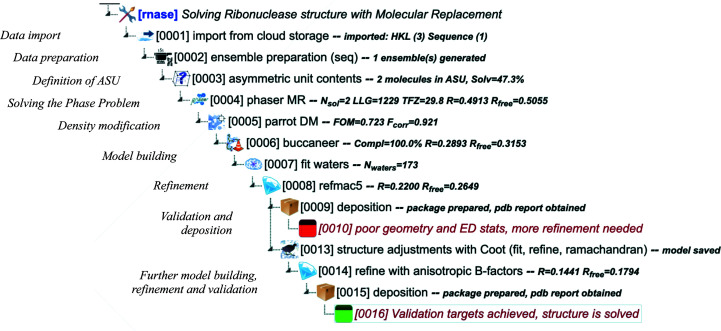
Screenshot of a *CCP*4 Cloud project. An indication of the principal structure solution stages is displayed on the left. See the discussion in the text.

*CCP*4 Cloud projects (exemplified in Fig. 4 ▸) are developed by adding Jobs to the nodes of the Project Tree. This should be performed while being mindful of the visibility of produced data objects for subsequent Jobs, as described above. If data objects required for a Job are not found in the current branch of the tree, then such a Job cannot be added. Typically, *CCP*4 Cloud projects contain a few stages.

#### 3.3.1. Data import

Data files may be uploaded to *CCP*4 Cloud from a local machine or imported from Cloud Storage. The main function of these tasks is to check the validity of files and create associated data objects. Diffraction images are not uploaded to *CCP*4 Cloud due to their considerable size. Instead, they are processed locally on the CC with *xia*2, *DUI*, *iMosflm* or *XDSGUI*, and the resulting reflection data sets are imported automatically at the end.

#### 3.3.2. Data preparation

This stage includes tasks such as scaling and merging reflections imported from unmerged MTZ files with *AIMLESS* (Evans & Murshudov, 2013 ▸) and the preparation of models for molecular replacement using *MrParse* (Simpkin *et al.*, 2022 ▸), *MrBUMP*, *CCP*4*mg* and similar.

#### 3.3.3. Definition of the asymmetric unit

This task sets the expected content of the asymmetric unit and performs Matthews analysis (Matthews, 1968 ▸). As a result, the initial Structure Revision is created, containing only the reflection data set and asymmetric unit composition. Having an initial­ized Structure Revision is necessary to proceed to the following stages.

#### 3.3.4. Solving the phase problem

Initial phases may be found with either molecular replacement or experimental phasing, or their combination. *CCP*4 Cloud includes both automatic structure solvers (*MrBUMP*, *MoRDa*, *BALBES*, *ARCIMBOLDO* and *Crank*2) and fundamental tasks (*SHELX*, *Phaser* and *MOLREP*; Vagin & Teplyakov, 2010 ▸). Obtained approximations for phases and atomic coordinates are added to the Structure Revision to be improved in subsequent stages.

#### 3.3.5. Density modification

*CCP*4 Cloud includes several tasks for density improvement: *Parrot* (Cowtan, 2010 ▸), *SHELXE* (Usón & Sheldrick, 2018 ▸) and *ACORN* (Yao *et al.*, 2005 ▸). These tasks result in a new version of the Structure Revision in which the density map(s) are replaced accordingly.

#### 3.3.6. Model building and refinement

Usually, the structure is built automatically in the first attempt using one of a few available tools: *Buccaneer* (Cowtan, 2006 ▸), *ARP*/*wARP*, *CCP*4*Build* (Krissinel & Lebedev, unpublished work) and *ModelCraft* (Bond & Cowtan, 2022 ▸). However, ultimately the structural model will require multiple manipulations with *REFMAC* and *Coot*. The process is highly iterative, and after running each model-building or refinement task a new Structure Revision is created that contains the improved atomic coordinates and electron-density maps.

#### 3.3.7. Validation, analysis and deposition

At the end of the structure-solution process, atomic coordinates from the last Structure Revision, along with reflection data, sequences and ligand descriptions, should be deposited in the Protein Data Bank (PDB; Berman *et al.*, 2000 ▸). Deposition files, in mmCIF format, are prepared with a dedicated *CCP*4 Cloud task. However, before deposition, and iteratively during the model-building and refinement procedure, the structure should be validated using the tools available in *Coot* and by inspection of the Quality Assessment section in the Job report for the *REFMAC* task. Furthermore, the space-group hypothesis may be checked using *Zanuda*, carbohydrate structures are validated with *Privateer* (Agirre *et al.*, 2015 ▸) and additional analysis and validation may be performed with *PISA*.

*CCP*4 Cloud Projects are objects themselves that can be exported as single files (ZIP archives), encapsulating all Jobs, data and metadata. The exported Projects are cross-platform compatible with all Microsoft Windows, Linuxes and Mac OS X. They can be re-imported in any instance of *CCP*4 Cloud; for example, that running on the user’s own machine. Note that importing/exporting Projects is different from project sharing, which is described below. The main purpose of this facility is to mitigate limitations of the disk allocation quota given to users in centralized installations of *CCP*4 Cloud and also to serve as an additional backup. For example, in the *CCP*4 Cloud instance maintained at RAL Harwell, disk space is initially capped at 15 GB (configurable). If all of the space is consumed, no new Jobs can be created. By exporting completed Projects and deleting them in *CCP*4 Cloud, their disk space is released back to the user. If a Project size grows beyond the initial allocation, the disk quota can be increased by a *CCP*4 Cloud administrator per user request. There is no expiry date for Projects in *CCP*4 Cloud.

## 4. *CCP*4 Cloud as a community resource

CCP4 has a mission to facilitate research in MX primarily by maintaining, developing and distributing the integrated *CCP*4 software suite, but also by educating and training scientists in experimental structural biology and via the wide dissemination of new ideas, techniques and practices. *CCP*4 Cloud fits the CCP4 mission very neatly.

### 4.1. Integration of third-party resources and software

*CCP*4 Cloud is designed as a highly synergetic platform for the integration of software components, databases and web services from CCP4 and other projects. The system includes generic frameworks for importing data from external sources [for example, the wwPDB (Berman *et al.*, 2003 ▸) and the AlphaFold database (Varadi *et al.*, 2022 ▸)] and running programs that are not included in the *CCP*4 software suite, such as *XDS* (Kabsch, 2010 ▸) and *XDSGUI*, *OpenFold* (Ahdritz *et al.*, 2021 ▸) or *AlphaFold*2 (Jumper *et al.*, 2021 ▸) for structure prediction, and *BUSTER* from Global Phasing Ltd (Bricogne *et al.*, 2017 ▸) for model refinement. The frameworks include mechanisms for requesting or validating user licences online (for example, authorization from Global Phasing Ltd is required before the *BUSTER* task is made available to a user). By assembling complex software in an integrated and harmonized system with 24/7 availability, *CCP*4 Cloud facilitates the delivery of novel methods and developments to a wide structural biology community.

### 4.2. Teamwork

*CCP*4 Cloud provides a convenient environment for organizing and storing structure-solution projects conducted by a research group or institution. Once in *CCP*4 Cloud, the projects are automatically backed up, and their lifetime is only limited by local data policies and IT support. All metadata in *CCP*4 Cloud are versioned and designed to be backward compatible: completed projects can therefore be revisited and revised or extended at any point in the future. *CCP*4 Cloud projects can be shared between two or more *CCP*4 Cloud users. In this way, several researchers can work on a project simultaneously and see each other’s actions in real time. This unique feature is particularly convenient for distributed teamwork, exchange between collaborating organizations, training young scientists and reporting software bugs. Subject to user consent, failed jobs with the corresponding input data can be automatically retained in a dedicated area and reported to the *CCP*4 Cloud maintainer. This mechanism, in combination with project sharing, was found to be extremely useful for software debugging and obtaining feedback from the research community, which is essential for further development of *CCP*4 software.

### 4.3. Documentation and educational capabilities

*CCP*4 Cloud documentation articles are written in RST format (https://docutils.sourceforge.io/rst.html) and placed in a dedicated GitLab repository. On modification, documentation is automatically compiled and moved into appropriate parts of the system, becoming linked with graphical interfaces. The documentation includes four main parts.

(i) *Task reference* contains articles related to specific tasks, focusing on their position in the overall structure-solution workflow. This includes method descriptions, which should be sufficient for the general understanding of the main parameters. Where possible, links to original publications and the author’s manuals are provided for in-depth investigation.
(ii) *User guide* aims at explaining the main principles of the *CCP*4 Cloud graphical interface.
(iii) *Developer reference* is available only to users tagged as ‘developer’ and contains setup instructions and a description of the framework for writing task interfaces.
(iv) *Tutorials* include examples of structure-solution projects demonstrating the use of structure-solution tech­niques in *CCP*4 Cloud, such as data processing, automatic and fundamental MR and experimental phasing, MR-SAD, refinement and model building *etc*. Tutorials are presented as seed projects with pre-loaded data, which users can import into their accounts. The user would then develop the project, following the instructions in the accompanying document.
(v) *CCP*4 Cloud documentation is set as a parallel project with moderated access for contributors, which makes it easy to adjust content for rapidly developing tasks, add new articles and make changes instantly available to end users. The tutorial framework is widely used at CCP4 MX schools and in some university courses. Any relevant tutorials with demo projects (including data) can be placed by participating contributors in *CCP*4 Cloud documentation to facilitate education in MX either on a local (for example a university) or a global level.

## 5. Discussion and conclusions

Although *CCP*4 Cloud does not currently expose all components of the *CCP*4 software suite through its graphical interface, it represents a sufficiently complete system for structure solution and includes all principal stages of MX computing from image processing to model deposition. A few alternatives exist for each stage, leading to functional redundancy but a higher probability of solving structures in difficult cases for the novice or experienced MX user. We therefore argue that *CCP*4 Cloud is functionally complete for use in the majority of practical cases. *CCP*4 Cloud is distributed as part of the *CCP*4 software suite (version 7.1 and higher) and may be used out of the box for working with the public instance of *CCP*4 Cloud maintained at RAL Harwell (https://cloud.ccp4.ac.uk). In addition, any *CCP*4 7.1+ setup may be configured as a server featuring a local instance of *CCP*4 Cloud, allowing users to benefit from in-house computational resources and avoid transmitting sensitive data to public servers.

*CCP*4 Cloud can be installed on a range of hardware platforms and operating systems (Linux, Mac OS X and MS Windows) and allows mixed setups including individual workstations, in-house clusters, centralized HPC facilities and generic clouds. *CCP*4 Cloud setups can be adjusted to a wide range of available resources; in the simplest configuration, *CCP*4 Cloud acts as a mere GUI on a single laptop or PC.

Designing a modern, data-driven interface was one of the main ambitions of the *CCP*4 Cloud project. *CCP*4 Cloud’s GUI eliminates the burden of managing many files, as are typically produced in MX projects, by abstracting them with composite data types and managing all data flows within *CCP*4 Cloud. MX projects are presented in the form of branched Project Trees, using a logically clear system for naming the data objects produced during the course of a project. Jobs are highlighted with summary statistics where appropriate to qualify or quantify Job success. Altogether, this visual representation makes projects quickly and easily navigable. Project development is made simple by way of growing the Project Tree via adding and removing Jobs with primitive graphical actions. Project branching is made unambiguous by introducing the concept of Structure Revisions, which can be considered as check points corresponding to specific states of the structure-solution workflow.

While *CCP*4 Cloud might be perceived as a mere web-based HPC-powered GUI for the *CCP*4 software suite, there are significant differences upon closer inspection. Firstly, the centralized online setup provides a natural basis for combining *CCP*4, web resources and third-party software. Examples include the PDB, the AlphaFold database at the EBI and software from Global Phasing and *AlphaFold*2. Secondly, *CCP*4 Cloud provides an environment for teamwork, where team members can be located anywhere but can work on the same projects, similar to other online systems such as Google Docs or GitHub. This feature is also important for education and training, as *CCP*4 Cloud can store virtually indefinite volumes of relevant documents and data integrated with demo projects. Thirdly, *CCP*4 Cloud provides a framework for the long-term storage of structure-solution projects and results, which is maintained to industry standards. This feature is important for research projects that span many years and helps data management in research laboratories and companies.

The significance of retaining as much data as possible cannot be overestimated. Quite often, structural experiments cannot be repeated under identical conditions, and misplaced data may not be recovered. Historically, the PDB aimed to keep only the final atomic coordinates, which is sufficient for most downstream studies. From 1999, merged reflection data could be deposited in addition to atomic coordinates; this became mandatory in 2008 (Burley *et al.*, 2017 ▸). This move was useful for the development of crystallographic software, but also allowed researchers to investigate structural data on a deeper level, permitting alternative interpretation of electron-density maps and further structure improvement in some cases (see, for example, *PDB-REDO*; Joosten *et al.*, 2009 ▸). The deposition of unmerged reflection data, which started in 2020, is another important step in this direction. Thus, the PDB is reaching a state where it captures experimental data in the form of indexed reflections and final interpretation in the form of an atomic model and electron-density maps. However, the PDB does not capture the way in which this interpretation was performed: information about the workflow and modelling decisions made during a structure-solution project is lost. We would like to advocate the idea that retaining structure-solution projects is as important as retaining experimental data. This can be useful for the subsequent understanding of ambiguous structural features, revisiting and revising structural studies, education, facilitating the peer-review publication process, training and software development. We further suggest that *CCP*4 Cloud provides a convenient framework for archiving structure-solution projects, complementing data repositories at the wwPDB, and we consider the implementation of such archival as one possible future direction.

The full power of *CCP*4 Cloud is unlocked when it is configured to receive data directly from synchrotron beamlines. The combination of MX facilities equipped with remote access, *CCP*4 Cloud and the wwPDB creates a closed online environment for macromolecular crystallography, which streamlines data logistics and maintenance. In its current implementation, *CCP*4 Cloud includes a facility to push data, such as reflections, sequences and models, to the user’s account and to then automatically create new structure-solution projects and start automatic structure-solution pipelines (see the details at https://cloud.ccp4.ac.uk/manuals/html-userguide/jscofe_cloudrun.html). In an ideal hypo­thetical situation, a user could send samples to a *CCP*4 Cloud-connected synchrotron and then simply check the results of automatic data processing and structure solution in their *CCP*4 Cloud account (for example on their smartphone or tablet). The success and practical significance of such a scheme will greatly depend on beamline automation and the performance of automatic structure-solution pipelines. Automatic methods have improved quite noticeably during the last decade, and it is expected that further advances will be associated with the availability of *AlphaFold*-predicted models for use in molecular replacement (Millán *et al.*, 2021 ▸; Pereira *et al.*, 2021 ▸; McCoy *et al.*, 2022 ▸; Medina *et al.*, 2022 ▸). In addition, the logically strict concepts behind project structure in *CCP*4 Cloud provide good potential for automating project development and adjusting to the variety of data properties and structural features. Progress towards the above targets represents yet another direction of future work.

## 6. Data access

Source code for *CCP*4 Cloud is included in the *CCP*4 software suite version 7.1 and higher, as obtainable from https://www.ccp4.ac.uk. It can also be found in GitLab repositories at https://gitlab.com/CCP4/jsCoFE. *CCP*4 Cloud documentation is maintained in GitLab at https://gitlab.com/CCP4/jscofe-doc. *CCP*4 Cloud and *CCP*4 Cloud documentation are provided free to academic users under the terms of the general CCP4 Licence (https://www.ccp4.ac.uk/licensing/academic_software_licence.pdf).

## Figures and Tables

**Table 1:** Data objects used in *CCP*4 Cloud See the discussion in Section 3.

Data object	ID†	File equivalent
Unmerged reflection data	UNMERGED	MTZ or HKL file with unmerged reflection data
Merged reflection data	HKL	Relevant columns of MTZ file with merged reflection data (*e.g.* *h*, *k*, *l*, *F*, sig*F*) or SCA file
Macromolecular sequence	SEQUENCE	Sequence files in FASTA or PIR format
Macromolecular coordinates	XYZ	PDB or mmCIF coordinate file
Phases	PHASES	Relevant columns of MTZ file (*e.g.* FWT, PHWT)
Ligand description	LIGAND	CIF file with ligand description
Ligand library	LIB	CIF or LIB file with multiple ligand descriptions
MR model	MODEL	Combination of sequence and single-model coordinate files
MR ensemble	ENSEMBLE	Combination of sequence and multi-model coordinate files
Structure	STRUCTURE	Combination of related XYZ and PHASES objects
Structure revision	REVISION	Combination of HKL, SEQUENCE and STRUCTURE objects

† Used for automatic naming of data objects in *CCP*4 Cloud.
